# Nosocomial lower respiratory tract infections in patients with immunosuppression: a cohort study

**DOI:** 10.1186/s13613-025-01462-y

**Published:** 2025-05-06

**Authors:** Luis Felipe Reyes, Natalia Sanabria-Herrera, Saad Nseir, Otavio T. Ranzani, Pedro Povoa, Emilio Diaz, Marcus J. Schultz, Alejandro Rodríguez, Cristian C. Serrano-Mayorga, Gennaro De Pascale, Paolo Navalesi, Szymon Skoczynski, Mariano Esperatti, Luis Miguel Coelho, Andrea Cortegiani, Stefano Aliberti, Anselmo Caricato, Helmut J. F. Salzer, Adrian Ceccato, Rok Civljak, Paolo Maurizio Soave, Charles-Edouard Luyt, Pervin Korkmaz Ekren, Fernando Rios, Joan Ramon Masclans, Judith Marin, Silvia Iglesias-Moles, Stefano Nava, Davide Chiumello, Lieuwe D. J. Bos, Antonio Artigas, Filipe Froes, David Grimaldi, Mauro Panigada, Fabio Silvio Taccone, Massimo Antonelli, Antoni Torres, Ignacio Martin-Loeches, for the European Network for ICU-Related Respiratory Infections (ENIRRIs) European Respiratory Society-Clinical Research Collaboration Investigators

**Affiliations:** 1 Unisabana Center for Translational Science, School of Medicine, Universidad de La Sabana, Chia, Colombia; 2 Clinica Universidad de La Sabana, Chia, Colombia; 3 Critical Care Department, Clinica Universidad de La Sabana, Chia, Colombia; 4 ISARIC, Pandemic Sciences Institute, University of Oxford, Oxford, UK; 5 University Hospital of Lille, Lille, France; 6 Barcelona Institute for Global Health, ISGlobal, Hospital Clínic-Universitat de Barcelona, Barcelona, Spain; 7 Pulmonary Division, Heart Institute (InCor), Hospital das Clinicas HCFMUSP, Faculdade de Medicina, Universidade de Sao Paulo, São Paulo, Brazil; 8 NOVA Medical School, NOVA University of Lisbon, Lisbon, Portugal; 9 Center for Clinical Epidemiology and Research Unit of Clinical Epidemiology, OUH Odense University Hospital, Odense, Denmark; 10 Intensive Care Unit 4, Dpt of Intensive Care, Hospital de São Francisco Xavier, CHLO, Lisbon, Portugal; 11 Corporacio Sanitaria Parc Tauli, Sabadell, Spain; 12 Intensive Care, Amsterdam UMC, University of Amsterdam, Amsterdam, The Netherlands; 13 Department of Intensive Care, Laboratory for Experimental Intensive Care & Anesthesiology (L E I C A), Amsterdam, The Netherlands; 14 Hospital Joan XXIII de Tarragona, Tarragona, Spain; 15 PhD Bioscience Engineering School, Universidad de La Sabana, Chia, Colombia; 16 Department of Intensive Care and Anesthesiology, Fondazione Policlinico Universitario A. Gemelli IRCCS Rome, Rome, Italy; 17 Magna Graecia University, Catanzaro, Italy; 18 Sant’Andrea (ASL VC), Vercelli, Italy; 19 Department of Lung Diseases and Tuberculosis, Faculty of Medical Sciences in Zabrze, Medical University of Silesia, Katowice, Poland; 20 Hospital Privado de Comunidad, Escuela Superior de Medicina, Universidad Nacional de Mar del Plata, Mar del Plata, Argentina; 21 Hospital de Sao Francisco Xavier, Lisbon, Portugal; 22 Department of Precision Medicine in Medical, Surgical and Critical Care (Me.Pre.C.C.), University of Palermo, Palermo, Italy; 23 Department of Anesthesia Intensive Care and Emergency, Policlinico Paolo Giaccone, Palermo, Italy; 24 Medical University of Silesia, Katowise, Poland; 25 Department of Biomedical Sciences, Humanitas University, Milan, Italy; 26 IRCCS Humanitas Research Hospital, Respiratory Unit, Milan, Italy; 27 Division of Infectious Diseases and Tropical Medicine, Department of Internal Medicine 4 - Pneumology, Kepler University Hospital, Linz, Austria; 28 Medical Faculty, Johannes Kepler University Linz, Linz, Austria; 29 Ignaz Semmelweis Institute, Interuniversity Institute for Infection Research, Vienna, Austria; 30 Critical Care Center, Hospital Universitari Parc Taulí, Institut d’Investigació I Innovació Parc Taulí (I3PT-CERCA), Sabadell, Spain; 31 Centro de Investigación Biomédica en Red en Enfermedades Respiratorias (CIBERES), Instituto de Salud Carlos III, Madrid, Spain; 32 Intensive Care Unit, Hospital Universitari Sagrat Cor, Grupo Quironsalud, Barcelona, Spain; 33 Intensive Care Department, Hospital Universitari Vall d’Hebron, Vall d’Hebron Institut de Recerca, Barcelona, Spain; 34 University of Zagreb School of Medicine, Zagreb, Croatia; 35 Sorbonne Université, Service de Médecine Intensive Réanimation, Groupe Hospitalier Pitié-Salpêtriere, Assistance Publique–Hôpitaux de Paris, Paris, France; 36 Ege University Medical Faculty, Izmir, Turkey; 37 Hospital Nacional Alejandro Posadas, Buenos Aires, Argentina; 38 Critical Care Department, Hospital del Mar, GREPAC, Hospital del Mar Research Institute, MELIS, Universitat Pompeu Fabra, Barcelona, Spain; 39 Hospital del Mar, Barcelona, Spain; 40 Hospital Arnau de Vilanova de Lleida, Lleida, Spain; 41 Alma Mater Studiorum, Department of Medical and Surgical Sciences (DIMEC), University of Bologna, 40138 Bologna, Italy; 42 Respiratory and Critical Care Unit, IRCCS Azienda Ospedaliero Universitaria di Bologna, Sant’Orsola Hospital, Bologna, Italy; 43 ASST Santi Paolo e Carlo, Milan, Italy; 44 Intensive Care Medicine Department, Corporacion Sanitaria Universitaria Parc Tauli, Institut d´Investigació I Innovació Parc Tauli I3PT, CIBER Enfermedades Respiratorias, Autonomous University of Barcelona, Sabadell, Spain; 45 Chest Department, Hospital Pulido Valente, CHULN, Lisbon, Portugal; 46 Hopital Universitaire de Bruxelles (HUB), Université Libre de Bruxelles (ULB), Brussels, Belgium; 47 Anesthesia and Critical Care, Fondazione IRCCS Ca’ Granda Ospedale Maggiore Policlinico, Milan, Italy; 48 Hospital Clinic of Barcelona, Barcelona, Spain; 49 James’s University Hospital, Trinity College, Dublin 8, D08 NHY Ireland

**Keywords:** Immunosuppression, Nosocomial lower respiratory tract infections, Critical care

## Abstract

**Background:**

This post-hoc analysis of a multinational, multicenter study aimed to describe and compare clinical characteristics, microbiology, and outcomes between immunosuppressed and non-immunosuppressed patients with nosocomial lower respiratory tract infections (nLRTI). The study utilized data from the European Network for ICU-related Respiratory Infections, including 1,060 adult ICU patients diagnosed with nLRTI. Descriptive statistics were used to compare baseline characteristics and pathogen distribution between groups. A Cox proportional hazards model stratified by immunosuppression status was applied to assess 28-day mortality risk, adjusting for disease severity and key clinical variables.

**Results:**

Immunosuppression was observed in 24.9% (264/1060) of the patients, and oncological conditions were the most common etiology of immunosuppression. Chronic pulmonary and cardiovascular diseases were the most frequent comorbidities. In both groups, *Pseudomonas aeruginosa* was the predominant microorganism, particularly affecting patients with immunosuppression (25.3% vs. 16.7%, p = 0.032). Cox regression model adjusted for disease severity (SAPS II), polytraumatized status, altered consciousness, and postoperative status, SAPS II remained a strong independent predictor of mortality, with each one-point increase associated with a 2.3% higher risk of death (HR: 1.023, 95% CI 1.017–1.030, p < 0.001). The analysis also revealed significant heterogeneity in mortality risk among immunosuppressed patients, with hematological malignancies, recent chemotherapy, and bone marrow transplantation associated with the highest mortality.

**Conclusions:**

Immunosuppressed patients had a lower adjusted survival probability compared to non-immunosuppressed patients. Moreover, *P. aeruginosa* was the most frequently identified etiological pathogen in immunosuppressed patients.

**Supplementary Information:**

The online version contains supplementary material available at 10.1186/s13613-025-01462-y.

## Introduction

Nosocomial lower respiratory tract infections (nLRTIs), particularly those associated with mechanical ventilation (VA-LRTI), are the most common infectious complications in the intensive care unit (ICU) [1]. These infections increase morbidity and have been linked to higher mortality rates in patients requiring invasive ventilation, particularly among extreme age groups [2]. Healthcare infections reach an annual burden in the US of $ 9.8 billion [3], and nLRTIs represent 31.7% of the total burden associated with healthcare-associated infections [3, 4]. Additionally, nLRTIs prolong hospital stays by nine days, increasing the attributable costs per case by $18,200 [3]. An economic burden analysis in China found that the median hospital costs for patients with nLRTIs were significantly higher than those without infections, resulting in an average additional cost per patient of $2,853.93 [5].

Patients with immunosuppression are particularly susceptible to severe infections with high fatality rates, especially when mechanical ventilation is required due to acute respiratory failure [4, 5]. The group of patients with immunosuppression encompass individuals undergoing chemotherapy for cancer, post-transplant recipients, those with acquired immunodeficiency syndrome (AIDS), and individuals taking steroids for autoimmune conditions. Advances in treating these conditions have extended the lives of affected patients, increasing the population of individuals with compromised immune systems. Acute respiratory failure contributes to poor outcomes due to an increased risk of opportunistic infections [6], greater disease severity, and poor functional status [1, 7]. However, the clinical characteristics and outcomes of immunosuppressed and non-immunosuppressed patients with nLRTIs may differ significantly and remain poorly characterized in the literature [1, 6, 7]. Most existing data on immunosuppressed patients with nLRTIs in the ICU derive from large cohorts of patients with acute respiratory failure, which may not fully capture the unique challenges of this specific population. Further research in immunosuppressed patients with nLRTIs is indicated, given the significant morbidity and mortality burden [1], to enhance mitigation strategies on par with the maximum clinical management of the condition. A deeper understanding in this regard would help develop a strategy that best reduces adverse events and improves the outcomes of critically ill patients due to nosocomial respiratory infections.

Thus, this multinational cohort study aims to characterize ICU patients with nLRTIs, comparing the clinical characteristics, microbiology, and outcomes of immunosuppressed and non-immunosuppressed patients. The primary objective is to assess the impact of immunosuppression on survival while adjusting for initial clinical severity. We hypothesize that patients with immunosuppression in the ICU with nLRTI, regardless of the specific cause of immunosuppression, have worse outcomes than non-immunosuppressed patients. By broadening the scope beyond Ventilator-Associated Pneumonia (VAP), this study provides a more comprehensive understanding of the burden of other nLRTIs in this high-risk population.

## Methods

This analysis used data from the prospective, multicenter, and multinational cohort study, the European Network for ICU-related Respiratory Infections (ENIRRI). This cohort includes critically ill patients admitted to 28 different intensive care units across 13 countries in Europe and South America, identified from May 9th, 2016, to August 16th, 2019. Approval for the study was obtained from the institution's Internal Review Board (Comité Ètic d'Investigació Clínica, registry number HCB/2020/0370), and informed consent was obtained from patients per local regulations. Subsequently, all clinical data was anonymized and transferred to the coordinating center for curation and analysis. This study was registered on ClinicalTrials.gov under the identifier NCT03183921. The study protocol was published and shared with researchers from each participating center before recruitment; further information about the study can be found elsewhere [6].

### Data collection

Patient data were collected from individuals meeting the following criteria: (1) aged over 18 years, (2) admitted to the ICU, and (3) diagnosed with nLRTIs following international guidelines and the study protocol [6]. Patients readmitted to the intensive care unit during the study collection period were excluded. The collected data encompassed demographic information, clinical characteristics, comorbidities, time of diagnosis, treatment received, laboratory and microbiological data, complications during ICU hospitalization, and outcomes. Disease severity was assessed using the Simplified Acute Physiology Score (SAPS) II and Sequential Organ Failure Assessment (SOFA) scores [7, 8].

### Definitions

According to the 2019 ATS/IDSA guidelines [9], pneumonia is defined by new lung infiltrates, clinical signs suggesting an infectious cause, recent onset of fever, purulent sputum, leukocytosis, and reduced oxygen levels [9]. In this study, nosocomial pneumonia is categorized as follows, based on contemporary definitions: hospital-acquired pneumonia (HAP) is defined as pneumonia that was not present or incubating at the time of hospital admission and develops 48 h or more after being admitted to the hospital; VAP is pneumonia that arises more than 48 h after invasive mechanical ventilation [10]. Ventilator-associated tracheobronchitis (VAT) is a lower respiratory infection of patients who received mechanical ventilation for at least 48 h but did not present with new radiographic infiltrates [11, 12]. ICU hospital-acquired pneumonia (ICU-HAP) refers to a lower respiratory tract infection acquired at least ≥ 48 h after the ICU admission, not requiring invasive mechanical ventilation due to the LRTI [6]. A ventilated HAP (VHAP) is defined as an nLRTI that develops outside the ICU at least 48 h after hospital admission and requires invasive mechanical ventilation due to the infection.

In this study, patients with immunosuppression were defined as those with active neoplasia, hematological malignancies, acquired immunodeficiency syndrome (AIDS), recipients of allogeneic stem cell or solid organ transplants, or those receiving immunosuppressive medications, including corticosteroids at doses > 30 mg/day for more than 30 days [1, 13–16]. For instance, we constructed an 'immunosuppressed' variable that includes the mentioned conditions for the analysis.

### Study groups

All consecutive patients diagnosed with nLRTIs were stratified into two groups within the cohort: those with a diagnosis of immunosuppressed or a history of immunosuppression due to medication (i.e., immunosuppressed patients) and those without such a diagnosis or history (i.e., non- immunosuppressed patients).

### Study outcomes

Our primary objective is to characterize the cohort of patients with immunosuppression diagnosed with nLRTIs admitted to the ICU. Additionally, we aim to evaluate the effect of immunosuppression on clinical outcomes, particularly in-hospital mortality, and compare the microbiological profiles between immunosuppressed and non-immunosuppressed patients.

### Statistical analysis

Continuous variables were described utilizing the median and interquartile range (IQR), while dichotomous variables were expressed in absolute and relative frequencies. Missing data were addressed, accounting for approximately 13–15% of the data in the variables of interest. Multiple imputation was conducted using the linear regression method to mitigate this issue. This approach allowed for the estimation of missing values based on the relationships observed in the existing dataset, enhancing the completeness and robustness of the analytical process.

For the univariate analysis, demographic variables and comorbidities were compared between the immunosuppressed and non-immunosuppressed groups to determine heterogeneity. Differences between the groups were assessed using the Chi-square test for categorical variables and the student's T-test for or the Mann–Whitney U test for continuous variables, depending on their distribution. Variables included in the univariate analysis were selected based on clinical relevance and potential confounding influence in the studied outcome. We grouped clinical variables into specific categories based on common characteristics to simplify the analysis and improve data interpretation. The cardiovascular category included myocardial infarction, congestive heart failure, and peripheral vascular disease. The neurologic category comprised dementia, paraplegia, and cerebrovascular disease. Variables related to liver function were divided into mild liver disease and severe liver disease. The renal category focused on chronic kidney disease. Finally, diabetes variables were grouped into diabetes with and without chronic complications. This classification allowed for a more structured and precise evaluation of the comorbidities present in the studied patients.

Kaplan–Meier curves and the Cox proportional hazards model were used to analyze time-to-event distributions for 28-day mortality following nLRTI diagnosis, adjusting for variables that were statistically significant in the univariate analysis (p value < 0.05). These factors were then incorporated into a multivariable Cox regression model, which was stratified by immunosuppression status to account for potential differences in baseline hazard functions between the two groups. Model selection was guided by the Akaike Information Criterion (AIC), ensuring optimal balance. Additionally, a subgroup analysis was conducted to evaluate the impact of specific immunosuppressive conditions on 28-day mortality. A separate Cox proportional hazards model was applied for each immunosuppressive subgroup, estimating hazard ratios (HRs) and 95% confidence intervals (CIs) while adjusting for relevant clinical covariates. The statistical analysis was performed using Stata software version 17 (StataCorp.2019) and RStudio Team version 2024.04 [17].

## Results

**Fig. 1:**
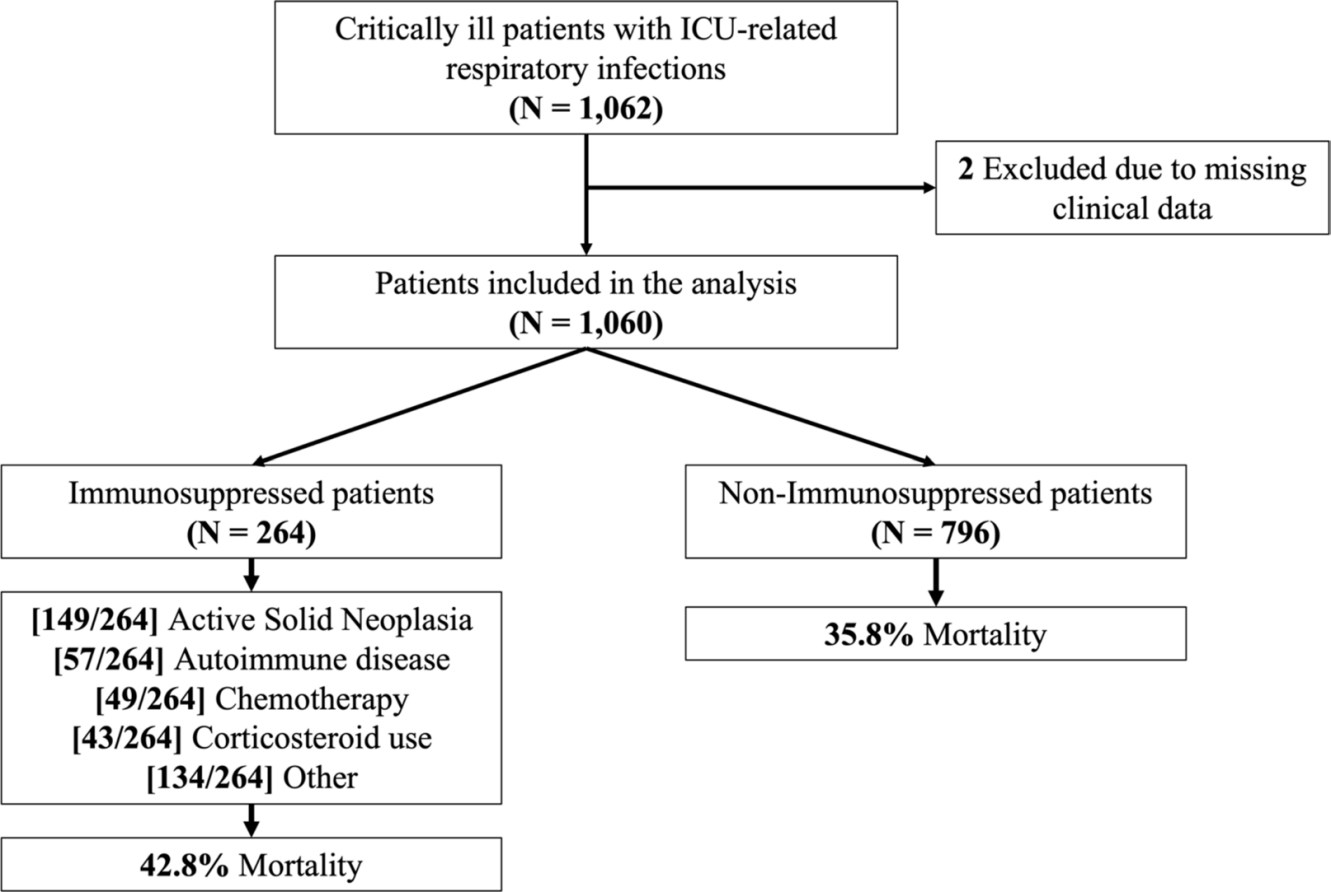
Study Flowchart. Flowchart of included patients diagnosed with ICU-related respiratory infections and clinical outcomes. *Please note that patients may have more than one state of immunosuppression; for further details, refer to Supplementary Fig. 1 and Supplementary Table 1, which provide additional information on this aspect

A total of 1,060 patients admitted to the ICU were included in the present study, with 25% (264/1060) classified in the immunosuppressed group, primarily due to non-metastatic solid tumors and autoimmune diseases (Fig. 1, Supplementary Table 1). Most of the patients enrolled were male (73%), with a median [IQR] age of 64 years [50–74] (Table 1). Before LRTI diagnosis, 44% (467/1060) of patients had a history of invasive mechanical ventilation, with a significantly higher proportion in the immunosuppressed group compared to the non-immunosuppressed group (50% vs. 42.1%, p = 0.025). The median hospital length of stay before LRTI diagnosis was 8 days (IQR: 4–16) overall but was significantly longer in immunosuppressed patients compared to non-immunosuppressed patients (11 days [IQR: 5–20] vs. 7 days [IQR: 4–14], p < 0.001). Similarly, the ICU length of stay before diagnosis differed between groups, with a median of 4 days (IQR: 1–9) overall, but a shorter duration in immunosuppressed patients (3 days [IQR: 0–10] vs. 5 days [IQR: 2–9], p = 0.008) (Table 1).

**Table 1:** Comparison of demographics, comorbidities, and outcomes between patients with and without immunosuppression

	Overall (n = 1060)	Immunosuppression risk factors (n = 264)	No- Immunosuppression risk factors (n = 796)	p-value
Demographics				
Age, median (IQR)	64 (50–74)	65 (54 -73)	64 (49–75)	0.34
Gender, male (%)	769 (72.5)	181 (68.6)	588 (73.9)	0.09
BMI, median (IQR)	25.9 (23.0–29.5)	25.0 (22.3–27.8)	26.2 (23.4–30.4)	**< 0.001**
Comorbidities (%)				
Diabetes	215 (20.3)	49 (18.6)	166 (20.9)	0.42
Chronic renal failure	120 (11.3)	28 (10.6)	92 (11.5)	0.67
Chronic heart disease	286 (26.9)	60 (22.7)	226 (28.4)	0.072
Chronic liver disease	66 (6.2)	18 (6.8)	48 (6.0)	0.65
Chronic lung disease	239 (22.5)	72 (27.2)	167 (20.9)	**0.03**
Drug abuse	208 (19.6)	47 (17.8)	161 (20.2)	0.39
Before LRTI diagnosis				
Previous Intubation (%)	467 (44)	132 (50)	335 (42.1)	**0.025**
Duration of Intubation, median days (IQR)	7 (2–14)	4 (0–13)	8 (3–14)	**0.001**
Previous Non-invasive Intubation (%)	151 (14.2)	45 (17)	106 (13.3)	0.133
Hospital length of stay until diagnosis, median days (IQR)	8 (4–16)	11 (5–20)	7 (4–14)	**< 0.001**
ICU length of stay until diagnosis, median days (IQR)	4 (1–9)	3 (0–10)	5 (2–9)	**0.008**
Type of LRTI (%)				
VAP	556 (52.5)	114 (43.2)	442 (55.5)	**< 0.001**
HAP	246 (23.2)	87 (32.9)	159 (20)	**< 0.001**
VAT	160 (15.1)	29 (10.9)	131 (16.5)	**0.031**
ICUHAP	98 (9.3)	34 (12.9)	64 (8.1)	**0.019**
Severity at ICU admission				
SAPS II, median (IQR)	47 (36–58)	44.5 (34–58)	47 (37–58)	0.29
SOFA, median (IQR)	7 (5–10)	8 (5–10)	7 (5–10)	0.61
Baseline Laboratories, median (IQR)				
PaO2/FiO2	194 (134–280)	177 (104–289)	200 (138–278)	**0.057**
Leucocytes	12.9 (9–18.5)	12.0 (6.3–19.0)	13.1 (9.4–18.2)	**0.015**
Outcomes				
Hosp LOS, median (IQR)	38 (22–65)	42 (25–70.5)	36.5 (21–63.5)	**0.018**
ICU LOS, median (IQR)	20 (11–35)	20 (11–35)	20 (11–35)	0.83
Death (%)	398 (37.5)	113 (42.8)	285 (35.8)	**0.042**
Cause of Death was MOF*	202 (19.1)	66 (25)	136 (17.1)	**0.005**
Cause of Death was Hipoxemia*	64 (6.0)	21 (7.9)	43 (5.4)	0.13
Death was attributable to LRTI*	81 (7.6)	27 (10.2)	54 (6.8)	0.068
Microbiological testing				
ETA-Sputum	513 (48.4)	132 (50)	381 (47.9)	0.547
BAL/miniBAL	620 (58.5)	163 (62.2)	457 (57.4)	0.216
Respiratory virus testing	134 (12.6)	48 (18.2)	86 (10.8)	**0.002**
Blood culture	832 (78.5)	212 (80.3)	620 (77.8)	0.408
Pleural fluid culture	52 (4.9)	28 (10.6)	24 (3.0)	**< 0.001**
PCR	87 (8.2)	28 (10.6)	59 (7.4)	0.101

*Cause of death was recorded by local investigators in the database based on predefined categories and was available only for patients who died in the ICU. These classifications were used as reported in the dataset without further adjudication

The most common nLRTIs diagnosed in both groups were VAP (556/1060; 53%), followed by HAP (152/1060; 14%). In patients with immunosuppression, VAP was the most frequent form of nLRTIs, accounting for 49% (94/194) of cases (Supplementary Table 2). The most frequent etiologies of immunosuppression were active solid neoplasia (56% [149/264]), autoimmune disease (22% [57/264]), chemotherapy (19% [49/264]), and chronic corticosteroid use (16% [43/264]) (Supplementary Fig. 1, Supplementary Table 1). The most frequent comorbidities in the immunosuppressed group were chronic lung diseases (72/264; 27%; p = 0.03) and chronic heart diseases (60/264; 23%; p = 0.07) compared to non-immunosuppressed patients.

The hospital length of stay was longer for patients in the immunosuppression group compared to the immunocompetent group (median [IQR]: 42 [25–70.5] days vs. 36.5 [21–63.5] days; p = 0.018). Additionally, a higher proportion of patients with immunosuppression died compared to non-immunosuppressed patients (43% [113/264] vs. 36% [285/796]; p = 0.042) (Table 1).

**Fig. 2:**
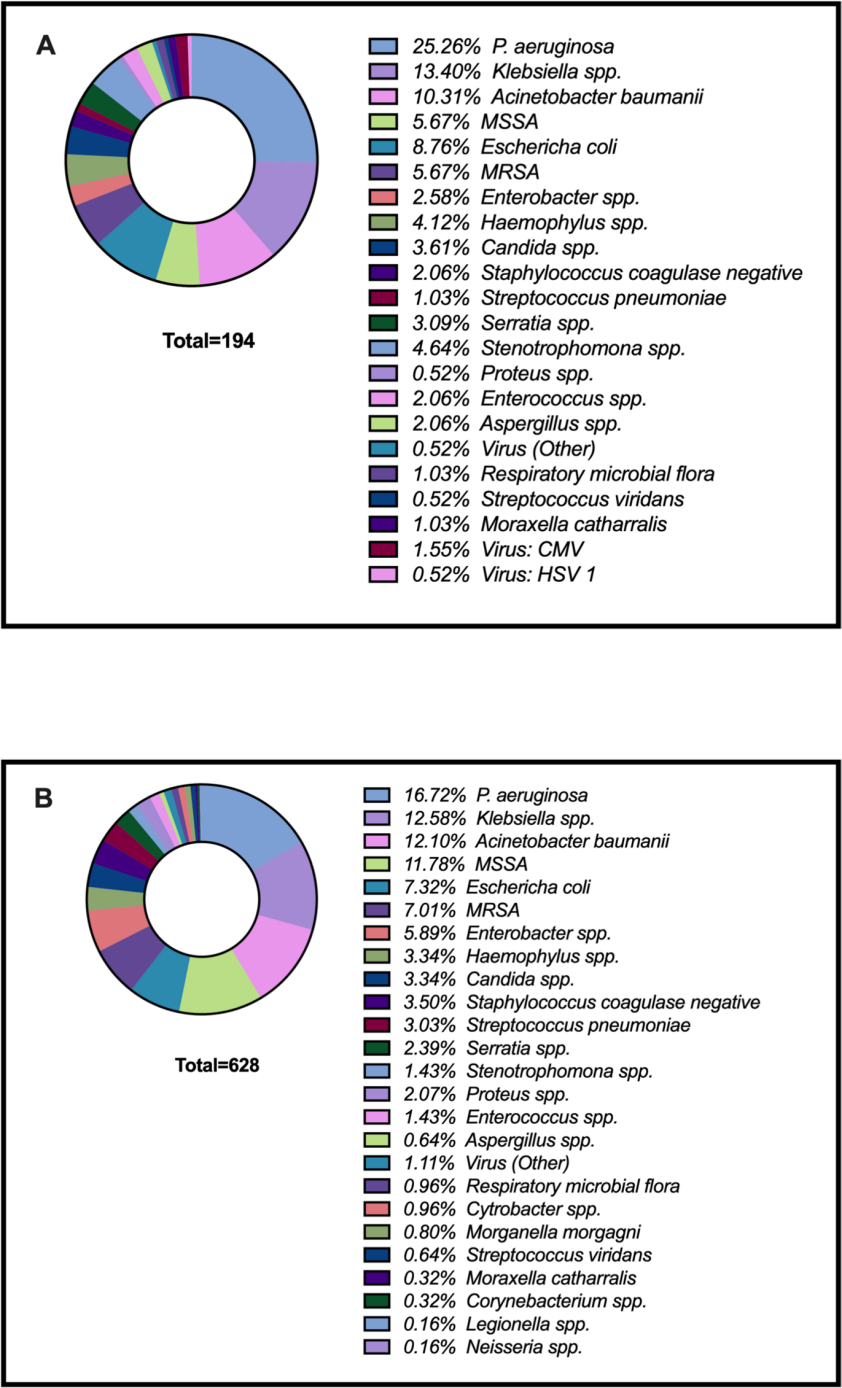
Identified microorganisms in **A** the Immunosuppressed and **B** the Non-Immunosuppressed group. Distribution of identified microorganisms in both immunosuppressed and non-immunosuppressed patients diagnosed with ICU-related respiratory infections. Specific pathogens and proportions are available in Supplementary Table 2

A total of 822 (78%) microorganisms were identified as the etiological agents of nLRTIs, and the prevalence of antibiotic resistance patterns was similar between immunosuppressed and non-immunosuppressed patients (Table 2). The causal agent was determined in 74% [194/264] of patients with immunosuppression and 79% [628/796] of non-immunosuppressed patients (p = 0.002). The most common pathogen in both groups was *Pseudomonas aeruginosa* (25% [49/154] vs. 17% [105/154]; p = 0.032), followed by *Klebsiella spp.* (13% [26/105] vs. 13% [79/105]; p = 0.97) and *Acinetobacter baumannii* (10% [20/96] vs. 12% [76/96]; p = 0.33) (Fig. 2). Among patients with immunosuppression, *P. aeruginosa* emerged as the predominant etiological agent in VAP cases, responsible for 37% (35/94) of infections. The main etiologies for other types of nLRTIs are detailed in Supplementary Table 2. Significant differences were found in respiratory virus testing (18% vs. 11%, p = 0.002) and pleural fluid culture (11% vs. 3%, p < 0.001), both being more common in patients with immunosuppression risk factors. Other testing methods, including ETA-sputum, BAL/miniBAL, blood cultures, and PCR, did not show statistically significant differences between the two groups (Table 1).

**Table 2:** Identified etiology stratified by immunosuppression status

Etiology (%)	Immunosuppression, n = 194	No-Immunosuppression, n = 628	p-value
Gram positive			
*MSSA*	11 (5.6)	74 (11.8)	**0.01**
*MRSA*	11 (5.6)	44 (7.0)	0.39
*Staphylococcus coagulase negative*	4 (2.1)	22 (3.5)	0.26
*Enterococcus* spp.	4 (2.1)	9 (1.4)	0.62
*Streptococcus pneumoniae*	2 (1.0)	19 (3.0)	0.1
Gram negative			
*Pseudomonas aeruginosa*	49 (25.3)	105 (16.7)	**0.032**
*Klebsiella* spp.	26 (13.4)	79 (12.6)	0.97
*Acinetobacter baumannii*	20 (10.3)	76 (12.1)	0.33
*Escherichia coli*	17 (8.7)	46 (7.3)	0.69
*Stenotrophomonas* spp.	9 (4.6)	9 (1.4)	**0.013**
Virus			
CMV	3 (1.5)	0 (0.0)	**0.003**
HSV 1	1 (0.5)	0 (0.0)	0.08
Virus (Other)	1 (0.5)	7 (1.1)	0.42
Antibiotic resistance patterns identified (%)			
MDR	38 (19.6)	94 (15.0)	0.27
ESBL	17 (8.8)	56 (8.9)	0.74
Carbapenemases resistant	17 (8.8)	39 (6.2)	0.332
XDR	11 (5.7)	41 (6.5)	0.521
PDR	3 (1.5)	4 (0.6)	0.271

The analysis of treatment regimens among patients with and without immunosuppression risk factors (Table 3) revealed that Piperacillin-tazobactam was the most frequently administered antibiotic overall, with no significant difference between the two groups (30.7% [81/264] vs. 27.1% [216/796]; p = 0.266). However, meropenem/imipenem was used more frequently in the immunosuppression group compared to those without immunosuppression (22.3% [59/264] vs. 15.3% [122/796]; p = 0.009). Amoxicillin-clavulanate was significantly less utilized in patients with immunosuppression (1.1% [3/264] vs. 9.8% [78/796]; p < 0.001) as well as Levofloxacin (1.1% [3/264] vs. 4.6% [37/796]; p = 0.009). No statistically significant differences were observed for other antibiotics, indicating similar usage patterns across both patient groups (Table 3, Supplementary Table 3).

**Table 3:** Antibiotic treatment received stratified by immunosuppression status

Treatment	Total, n = 1060	Immunosuppression, n = 264	No-Immunosuppression, n = 796	p-value
Beta-lactams with beta-lactamase inhibitors				
Piperacillin-tazobactam	297	81 (30.7)	216 (27.1)	0.266
Amoxyciline-clavulanate	81	3 (1.1)	78 (9.8)	**< 0.001**
Carbapenems				
Meropenem/Imipenem	181	59 (22.3)	122 (15.3)	**0.009**
Ertapenem	2	0 (0.0)	2 (0.3)	0.415
Cephalosporins				
Ceftriaxone	42	9 (3.4)	33 (4.1)	0.595
Cefepime/Cefpirome	28	8 (3.0)	20 (2.5)	0.649
Glycopeptides				
Vancomycin	62	11 (4.2)	51 (6.4)	0.179
Teicoplanin	2	0 (0.0)	2 (0.3)	0.415
Fluoroquinolones				
Levofloxacine	40	3 (1.1)	37 (4.6)	**0.009**
Ciprofloxacine	31	9 (3.4)	22 (2.7)	0.591
Other antibiotics				
Linezolyd	68	20 (7.6)	48 (6.0)	0.374
Colistin	48	15 (5.7)	33 (4.1)	0.298

**Fig. 3:**
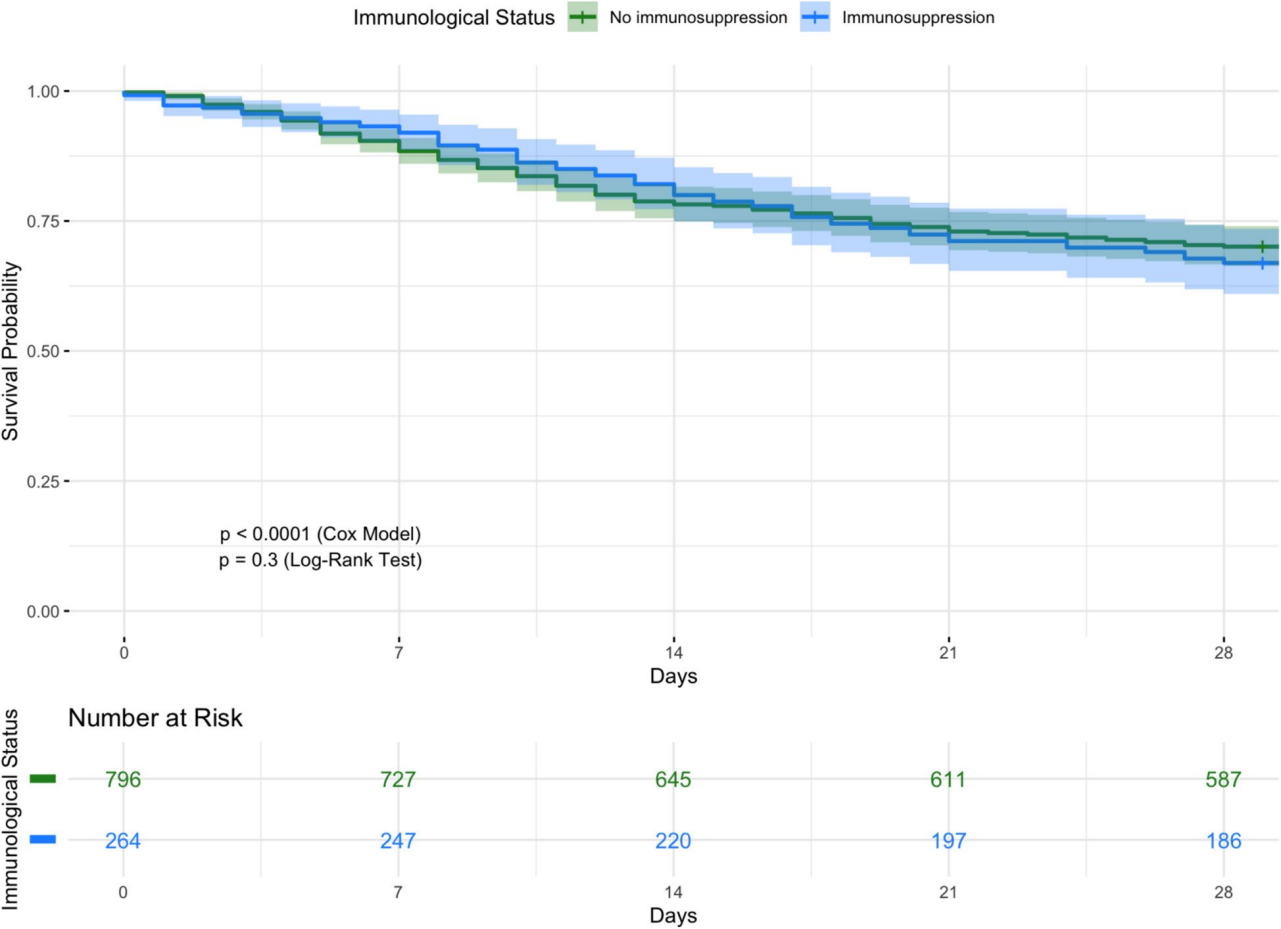
Kaplan–Meier Survival Curve at 28 days, Adjusted for Disease Severity Using SAPS II Score. Kaplan–Meier survival analysis comparing 28-day survival between immunosuppressed and non-immunosuppressed patients, adjusted for disease severity based on SAPS II scores

In the survival analysis, the Kaplan–Meier curve (Fig. 3) shows that patients with immunosuppression have significantly lower survival rates than non-immunosuppressed patients. The Cox regression analysis (Table 4), stratified by immunological status, revealed some key factors associated with 28-day mortality. A higher SAPS II score was strongly linked to an increased risk of death (HR 1.02, 95% CI 1.017–1.030, p < 0.0001), reinforcing its role as a predictor of severity. Interestingly, patients who were polytraumatized had a significantly lower risk of mortality (HR 0.17, 95% CI 0.06–0.46, p = 0.0004), suggesting that this group may have received more intensive monitoring and care. Similarly, both altered consciousness (HR 0.54, 95% CI 0.35–0.83, p = 0.0051) and postoperative status (HR 0.59, 95% CI 0.39–0.88, p = 0.0099) appeared to be protective factors, potentially reflecting differences in baseline health status or early intervention strategies. The subgroup analysis of immunosuppressed patients revealed significant variability in 28-day mortality risk depending on the underlying condition. Patients with active hematological neoplasia (HR: 1.98, 95% CI 1.19–3.30, p = 0.0086) and those who had recent chemotherapy within the last 30 days (HR: 1.93, 95% CI 1.07–3.48, p = 0.0285) showed a significantly higher mortality risk. The highest hazard ratio was observed in bone marrow transplant recipients (HR: 3.93, 95% CI 1.45–10.64, p = 0.0071), emphasizing their vulnerability (Supplementary Table 4).

**Table 4:** Multivariable cox regression model adjusted for immunological status

Variable	Coefficient (β)	HR (95% CI)	Standard Error	Z-score	p-value
SAPS II Score	0.023	1.02 (1.017–1.030)	0.003	7.04	**< 0.0001**
Polytraumatized	− 1.798	0.17 (0.06–0.46)	0.505	− 3.56	**0.0004**
Altered Consciousness	− 0.613	0.54 (0.35–0.83)	0.219	− 2.80	**0.0051**
Postoperative Status	− 0.536	0.59 (0.39–0.88)	0.208	− 2.58	**0.0099**

HR: Hazard Ratio, CI: Confidence Interval, SAPS II: Simplified Acute Physiology Score II, β (Coefficient): (Regression coefficient in the Cox model), SE: Standard Error, Z-score: Standardized test statistic used to assess statistical significance, and p-value: Probability value indicating statistical significance

In the multivariable analysis for in-hospital mortality, immunosuppression was associated with an increased risk of death in patients with nLRTI overall (OR 1.70 (1.10–2.62), p = 0.016) after adjusting for age and SAPS II at ICU admission. However, when stratified by nLRTI subtypes, this association was not significant in some subgroups. In patients with VAP, immunosuppression remained a significant predictor of mortality (OR 1.70 (1.10–2.62), p = 0.016). In contrast, in other subgroups (VAT, HAP, ICUAP, VHAP), immunosuppression was not significantly associated with in-hospital mortality (Supplementary Table 5). In these subgroups, other factors such as SAPS II or SOFA score at admission had a greater impact on clinical outcomes. Furthermore, the generally low Pseudo-R^2^ values across the models reflect that only a very small portion of variability in in-hospital mortality is explained by immunosuppression alone. The higher values were for HAP at 0.0806 and VHAP at 0.0838, suggesting an overall better fit of these subtypes within the model. VAT had a very low pseudo-R^2^ of 0.0193, indicating that other unmeasured factors are at play for VAT mortality. This speaks to heterogeneity among nLRTI subtypes.

## Discussion

Our results showed that oncologic conditions were our cohort's most common causes of immunosuppression. Chronic pulmonary and cardiovascular diseases were the most frequent comorbidities among these patients. The distribution of infection types observed in our study does not account for ICU patients who died before an nLRTI diagnosis. While HAP was the most frequent nLRTI in immunosuppressed patients, VAP, VAT, and ICU-HAP were more common in non-immunosuppressed group of patients. However, these findings should be interpreted considering survivorship bias, as patients who died early in their ICU stay—before developing nLRTI—were not included in the analysis. Regarding the microorganisms causing nLRTIs, *P. aeruginosa* was the most prevalent and strongly associated with immunosuppression. Finally, patients with immunosuppression had a lower adjusted survival probability than non-immunosuppressed patients.

Patients with immunosuppression have a lower survival probability compared to the non-immunosuppressed group of patients, a trend that aligns with prior research emphasizing elevated mortality risks in this vulnerable population [1, 18]. Unadjusted survival differences between these groups, however, were not statistically significant according to the log-rank test (p = 0.3), indicating that immunosuppression might not be the main mortality cause. The larger significance of total disease severity in patient outcomes was highlighted by our multivariable Cox regression model, which instead found SAPS II, polytraumatized state, altered consciousness, and postoperative status as independent predictors of 28-day mortality. Specifically, the heightened mortality observed in immunosuppressed individuals underscores the adverse effects of immunosuppression on clinical outcomes, especially when exacerbated by severe illness and elevated SAPS II scores [19–21]. The overall prevalence of mechanical ventilation was high in both groups (50% vs. 42.1%), despite disparities in exposure across groups, with immunosuppressed patients having a larger history of invasive ventilation. This suggests that ventilation status alone is insufficient to explain differences in survival fully. Interestingly, VAP was more common in immunocompetent individuals even though the immunosuppressed group had a higher history of prior intubation. This suggests that other factors, such as immune response, pathogen distribution, or antibiotic exposure, may affect outcomes. Additionally, our subgroup analysis showed that mortality risk varied significantly among immunosuppressed patients (Supplementary Table 4). We found that bone marrow transplantation, recent chemotherapy, and hematological malignancies were linked to a much higher risk of death, while autoimmune diseases and chronic corticosteroid use had no significant effect. These findings underscore the importance of a more precise risk stratification approach—one that goes beyond immunosuppression status to consider the underlying cause and severity of immune dysfunction in critically ill patients.

Despite improvements in ICU care, the persistently high mortality rates among patients with immunosuppression call for continued research and refined treatment protocols to improve survival outcomes for this critically ill population. Interestingly, some studies have found a lower incidence of VA-LRTI among immunosuppressed [1], which contrasts with the higher mortality observed in this analysis. The authors hypothesize that this discrepancy is likely linked to the higher exposure to previous antibiotic treatments in patients with immunosuppression, resulting in a higher rate of multidrug-resistant (MDR) VA-LRTI in this population.

*Pseudomonas aeruginosa* was the most frequently isolated pathogen in both immunosuppressed and non-immunosuppressed ICU patients, followed by *Klebsiella *spp*.* and *Acinetobacter baumannii*. This observation aligns with previous research, consistently identifying Gram-negative bacteria, particularly *P. aeruginosa* and *Acinetobacter baumannii*, as key microorganisms in ventilator-associated and nosocomial infections within ICU settings [18, 22]. Additionally, our results show a statistically significant presence of *P. aeruginosa* in the immunosuppressed group, with a p-value of 0.032, reinforcing the association between immunosuppression and the prevalence of this pathogen. The high prevalence of *P. aeruginosa* and *Klebsiella spp.* in our cohort mirrors results from Liu [23], who highlighted a similar trend among patients with immunosuppression. Despite these similarities, variability in microbial patterns across studies—such as the differing frequencies of specific pathogens [13, 22]—suggests that patient demographics and hospital environments significantly influence infection profiles. Given the higher prevalence of *P. aeruginosa* in immunosuppressed patients in this cohort, we suggest that early empirical antipseudomonal coverage should be considered in this population when managing nosocomial pneumonia [24, 25]. This recommendation should be tailored according to local epidemiology and institutional resistance patterns to ensure appropriate treatment.

Although bacterial pathogens were by far the most common etiologic agents identified in our cohort, both fungal-Candida spp., Aspergillus spp.-and viral infections-CMV, HSV-1-were detected (Supplementary Table 2). Generally, these opportunistic infections occur in highly immunocompromised patients and could complicate the clinical course, affecting outcomes [26, 27]. However, they were relatively infrequent in our data set, making further analysis more difficult. Future studies with larger cohorts and extending microbiological testing are required to delineate better the role of fungal and viral pathogens in nLRTI in immunocompromised patients.

The analysis of treatment regimens in patients with and without immunosuppression risk factors revealed significant differences in the use of certain antibiotics, highlighting the importance of adjusting antimicrobial therapy according to the patient's immunological status. Although piperacillin-tazobactam was the most frequently administered antibiotic, with no significant differences between the groups, using meropenem/imipenem was notably higher in patients with immunosuppression. This reflects the need for broad-spectrum antibiotics in this group due to their increased risk of severe and multidrug-resistant infections. These findings are consistent with previous studies that supported using meropenem combined with colistin because of the reduction of mortality risk, particularly for those with nLRTIs [28]. Additionally, the lower utilization of amoxicillin-clavulanate and levofloxacin in this group may be explained by concerns regarding their efficacy in the presence of resistant pathogens, as evidenced by recent analyses suggesting that empirical therapy in these patients should be more aggressive and focused on agents with a broad spectrum of activity. Interestingly, despite the increased use of broad-spectrum antibiotics like meropenem/imipenem in patients with immunosuppression, no significant differences were found in antibiotic resistance patterns between immunosuppressed and non-immunosuppressed patients, MDR organisms (19.6% vs. 15.0%, p = 0.27) and carbapenem-resistant strains (8.8% vs. 6.2%, p = 0.332). This suggests that while patients with immunosuppression are often treated with broader-spectrum antibiotics due to their higher risk, the prevalence of resistant infections in this cohort does not appear disproportionately higher [29].

The cohort's diversity, including across 13 countries, introduces potential variability in healthcare practices, diagnostic protocols, and treatment regimens. This may result in country-specific inclusion biases and potentially affect the study's generalizability. However, this diversity is also a strength, as it includes many patients and contexts. Furthermore, this may have influenced patient inclusion, introducing selection bias in the cohort. The study period from May 2016 to August 2019 also does not account for the COVID-19 pandemic, which has significantly altered respiratory infection dynamics and treatment practices. Other limitations include the decision-making process regarding life-sustaining therapies, which may influence patient outcomes. Given the observational nature of this study, both antibiotic selection and microbiological testing were determined by institutional protocols and clinician judgment rather than a standardized approach. However, adherence to ATS/IDSA guidelines was recommended. This variability in diagnostic practices and empirical antibiotic choices should be considered when interpreting our findings, as it may have influenced both pathogen identification and treatment strategies. For instance, the higher frequency of pleural fluid cultures and respiratory virus testing in immunosuppressed patients (10.6% vs. 3.0%, p < 0.001; 18.2% vs. 10.8%, p = 0.002, Table 1) suggests increased diagnostic intensity, which could introduce detection bias. Standardized diagnostic approaches are needed to ensure more accurate comparisons of infection rates and outcomes.

The presence of specialized versus non-specialized cancer and hematological ICUs could also impact treatment efficacy and patient management. Moreover, the study may not adequately differentiate attributable mortality due to LRTIs from mortality related to underlying diseases. There is considerable heterogeneity in immunosuppression among patients, as the effects of allogeneic bone marrow transplantation (alloBMT) differ significantly from those of chronic steroid therapy. In our study, we considered ‘patients with immunosuppression’ as one category, according to the definitions adopted from critical care and infectious disease literature. However, we must highlight that immunosuppression itself is not a homogeneous status, and the degree of immune dysfunction can vary substantially according to the main cause [26, 30]. A large percentage of the immunosuppressed patients in the cohort had active solid neoplasia without reporting chemotherapy, which may not show the same degree of immune depletion as hematologic malignancies, bone marrow transplantation, or chronic immunosuppressive therapy. While our findings indicate a higher burden of nLRTI in immunosuppressed patients, future studies should also consider stratification of immunosuppression subtypes to better define how specific conditions contribute to infection susceptibility and outcomes. Recognizing these differences is important to guide personalized prevention and management strategies in critically ill patients with immunosuppression.

## Conclusions

In conclusion, our study provides insights into the epidemiology and clinical outcomes of nLRTIs among immunosuppressed patients in the ICU. Immunosuppressed patients had a lower adjusted survival probability compared to non-immunosuppressed patients. The high prevalence of P. aeruginosa in this population highlights the challenges in when deciding empiric antimicrobial treatment in these patients, emphasizing the need for targeted treatment strategies. These findings underscore the importance of continued research to optimize the prevention and management of nosocomial infections in immunosuppressed critically ill patients.

## Supplementary Information

Supplementary Material 1. Supplementary Fig. 1. Upset plot representing the frequency of immunosuppression conditions. Illustrating the frequency and overlap of various immunosuppression conditions among the study population. Each set represents a specific combination of immunosuppression states, with bar heights indicating the number of patients in each category.

## Data Availability

The datasets analyzed during the current study are available from the corresponding author upon reasonable request.

## Abbreviations

AIDS: Acquired immunodeficiency syndrome
ATS: American Thoracic Society
ENIRRI: European Network for ICU-related Respiratory Infections
HAP: Hospital-acquired pneumonia
ICU: Intensive care unit
ICU-HAP: ICU hospital-acquired pneumonia
IQR: Interquartile range
nLRTI: Nosocomial lower respiratory tract infection
SAPS: Simplified acute physiology score
SOFA: Sequential organ failure assessment
VAT: Ventilator-associated tracheobronchitis
VA-LRTI: Ventilator-associated lower respiratory tract infection
VHAP: Ventilated hospital-acquired pneumonia
VAP: Ventilator-associated pneumonia
