# Supplementary material for: Nosocomial lower respiratory tract infections in patients with immunosuppression: a cohort study
Source: Ann Intensive Care. 2025 May 6;15:61. doi: 10.1186/s13613-025-01462-y (PMC12055687; doi:10.1186/s13613-025-01462-y)
Supplement: Supplementary file 1 — Supplementary Material 1. Supplementary Fig. 1. Upset plot representing the frequency of immunosuppression conditions. Illustrating the frequency and overlap of various immunosuppression conditions among the study population. Each set represents a specific combination of immunosuppression states, with bar heights indicating the number of patients in each category. [file 13613_2025_1462_MOESM1_ESM.docx]

**Supplementary Figure 1. Upset plot representing the frequency of immunosuppression conditions.**

**
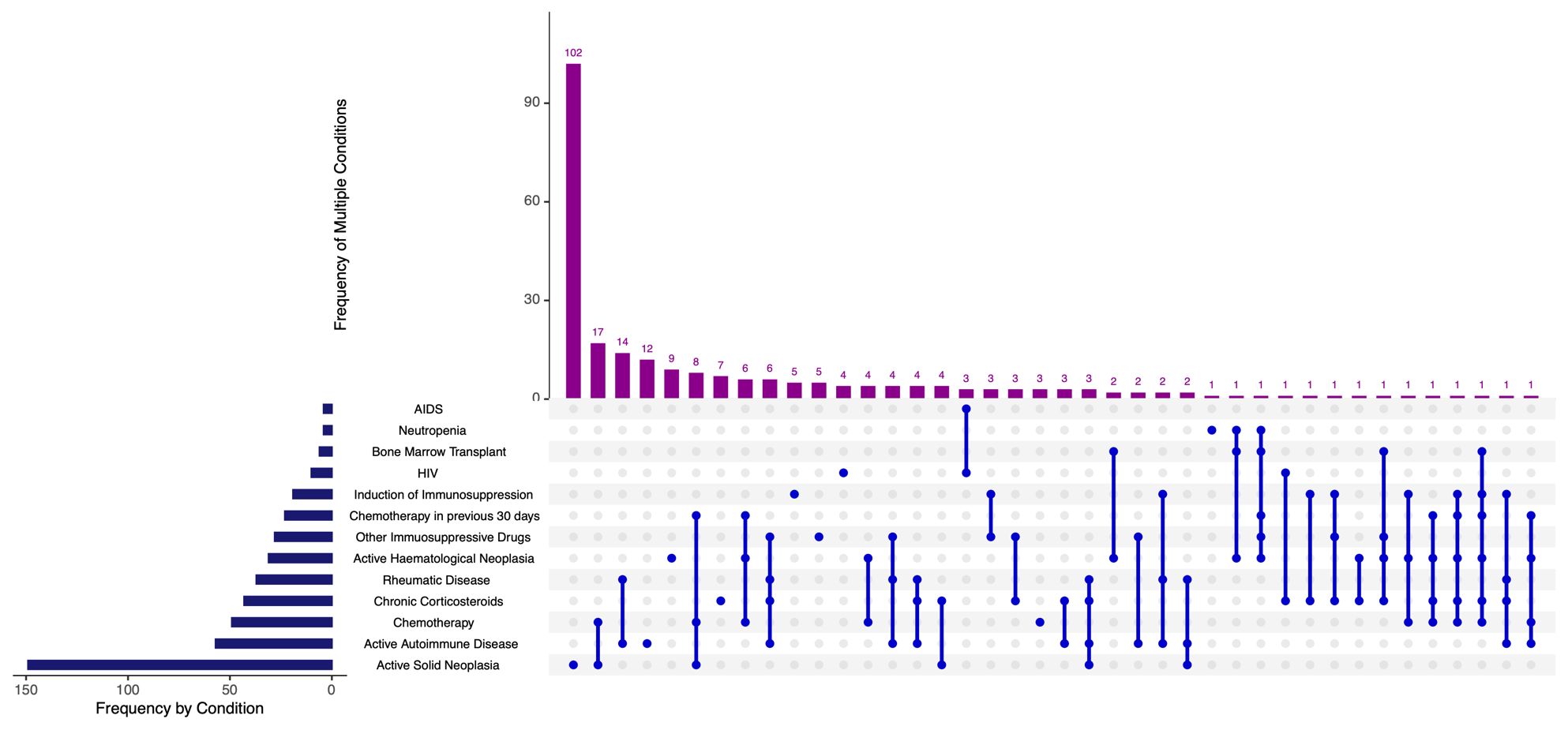
**

**Supplementary Table 1.** Types of Immunosuppression in Patients with nLRTIs Admitted to the ICU.

| **Types of Immunosuppression** | **n (%)** |
| --- | --- |
| Active solid neoplasia | 149 (14.1) |
| Autoimmune disease | 57 (5.38) |
| Chemotherapy | 49 (4.6) |
| Chronic corticosteroid use | 43 (4.1) |
| Rheumatic disease | 37 (3.5) |
| Active hematologic neoplasia | 31 (2.9) |
| Chemotherapy, last 30 days | 23 (2.2) |
| Induction of immunosuppression | 19 (1.8) |
| HIV | 10 (9.4) |
| Bone marrow transplant | 6 (0.6) |
| AIDS | 4 (0.4) |
| Neutropenia | 4 (0.4) |

**Abbreviations**: HIV, Human Immunodeficiency Virus; AIDS, Acquired Immunodeficiency Syndrome.

**Supplementary Table 2.** Identified microorganisms in patients with immunosuppression according to nLRTIs diagnosis

| **Etiology in Patients with Immunosuppression (%)** | **VAP,  n = 94** | **HAP,  n = 51** | **VAT, n = 27** | **ICUHAP,   n = 22** |
| --- | --- | --- | --- | --- |
| *Pseudomonas aeruginosa* | **35 (37.2)** | 4 (7.8) | **7 (25.9)** | **3 (13.6)** |
| *Klebsiella spp.* | 10 (10.6) | 6 (11.7) | **7 (25.9)** | **3 (13.6)** |
| *Acinetobacter baumannii* | 15 (15.9) | 4 (7.8) | 1 (3.7) |  |
| *Escherichia coli* | 6 (6.3) | **8 (15.7)** | 2 (7.4) | 1 (4.5) |
| *MSSA* | 7 (7.4) | 2 (3.9) | 1 (3.7) | 1 (4.5) |
| *MRSA* | 3 (3.2) | 5 (9.8) |  | **3 (13.6)** |
| *Stenotrophomonas spp.* | 4 (4.3) | 2 (3.9) | 1 (3.7) | 2 (9.0) |
| *Haemophilus spp.* | 3 (3.2) | 3 (5.8) | 1 (3.7) | 1 (4.5) |
| *Candida spp.* | 1 (0.6) | 6 (6.9) |  |  |
| *Serratia spp.* | 2 (2.1) |  | 4 (14.8) |  |
| *Enterobacter spp.* | 3 (3.2) | 2 (3.9) |  |  |
| *Staphylococcus coagulase negative* |  | 2 (3.9) |  | 2 (9.0) |
| *Enterococcus spp.* | 1 (0.6) |  | 2 (7.4) | 1 (4.5) |
| *Aspergillus spp.* |  | 3 (3.4) |  | 1 (4.5) |
| *CMV* |  | 1 (1.9) |  | 2 (9.0) |
| *Streptococcus pneumoniae* |  | 1 (1.9) | 1 (3.7) |  |
| *Respiratory microbial flora* | 1 (0.6) | 1 (1.9) |  |  |
| *Moraxella catarrhalis* | 1 (0.6) | 1 (1.9) |  |  |
| *Proteus spp.* | 1 (0.6) |  |  |  |
| *Virus (Other)* |  |  |  | 1 (4.5) |
| *Streptococcus viridans* | 1 (0.6) |  |  |  |
| *HSV 1* |  |  |  | 1 (4.5) |

**Abbreviations:** CMV: Cytomegalovirus**,** HAP: Hospital-acquired pneumonia, HSV: Herpes virus simplex, ICUHAP: Hospital-acquired pneumonia in the ICU, MSSA: Methicillin-Sensitive Staphylococcus aureus, MRSA: Methicillin-Resistant Staphylococcus aureus, VAP: Ventilator-associated pneumonia, VAT: Ventilator-associated tracheobronchitis.

**Supplementary Table 3.** Treatment given to patients stratified by immunosuppression status.

| **Treatment** | **Overall  n=1060** | **Immunosuppressed  n=264** | **No-Immunosuppressed**  **n=796** | ***p-value*** |
| --- | --- | --- | --- | --- |
| Piperacillin-tazobactam | 297 | 81 (30.7) | 216 (27.1) | 0.266 |
| Meropenem/Imipenem | 181 | 59 (22.3) | 122 (15.3) | **0.009** |
| Amoxicillin-clavulanate | 81 | 3 (1.1) | 78 (9.8) | **<0.001** |
| Linezolid | 68 | 20 (7.6) | 48 (6.0) | 0.374 |
| Vancomycin | 62 | 11 (4.2) | 51 (6.4) | 0.179 |
| Colistin | 48 | 15 (5.7) | 33 (4.1) | 0.298 |
| Ceftriaxone | 42 | 9 (3.4) | 33 (4.1) | 0.595 |
| Levofloxacine | 40 | 3 (1.1) | 37 (4.6) | **0.009** |
| Ceftazidime | 35 | 8 (3.0) | 27 (3.4) | 0.776 |
| Ciprofloxacine | 31 | 9 (3.4) | 22 (2.7) | 0.591 |
| Cefotaxima | 30 | 8 (3.0) | 22 (2.7) | 0.821 |
| Cefepime/Cefpirome | 28 | 8 (3.0) | 20 (2.5) | 0.649 |
| Amykacine | 27 | 9 (3.4) | 18 (2.3) | 0.305 |
| Cefazoline | 18 | 2 (0.8) | 16 (2.0) | 0.172 |
| Ampicilin | 17 | 2 (0.8) | 15 (1.8) | 0.207 |
| Cotrimoxazole | 10 | 5 (1.8) | 5 (0.6) | 0.065 |
| Beta-lactamic, other | 8 | 2 (0.8) | 6 (0.7) | 0.995 |
| Cloxacilline | 7 | 1 (0.4) | 6 (0.7) | 0.514 |
| Metronidazole | 3 | 1 (0.4) | 2 (0.3) | 0.735 |
| Gentamycine | 3 | 0 (0.0) | 3 (0.4) | 0.318 |
| Flucloxacillin | 3 | 2 (0.8) | 1 (0.1) | 0.094 |
| Ertapenem | 2 | 0 (0.0) | 2 (0.3) | 0.415 |
| Teicoplanin | 2 | 0 (0.0) | 2 (0.3) | 0.415 |
| Tigeciclina | 2 | 0 (0.0) | 2 (0.3) | 0.415 |
| Cephalosporin, other | 2 | 1 (0.4) | 1 (0.1) | 0.411 |
| Amphotericin B | 1 | 1 (0.4) | 0 (0.0) | 0.082 |
| Caspofungine | 1 | 1 (0.4) | 0 (0.0) | 0.082 |
| Cefuroxime | 1 | 0 (0.0) | 1 (0.1) | 0.565 |
| Erytromicin | 1 | 0 (0.0) | 1 (0.1) | 0.565 |
| NA | 4 | 1 (0.4) | 3 (0.4) | 0.997 |
| Antifungal, other | 2 | 0 (0.0) | 2 (0.3) | 0.415 |
| Fluconazole | 1 | 1 (0.4) | 0 (0.0) | 0.082 |
| Antiviral agents | 1 | 1 (0.4) | 0 (0.0) | 0.082 |
| Quinolones, other | 1 | 0 (0.0) | 1 (0.1) | 0.565 |
| **Abbreviations**: NA: Patients who did not receive any kind of antibiotic, antiviral, or antifungal treatment during the hospital stay or there is no record. | | | | |

**Supplementary Table 4.** Subgroup Analysis of Immunosuppressed Patients – Cox Proportional Hazards Model for 28-Day Mortality

| **Immunosuppessive condition** | **Sample Size** | **28-day mortality** | **Coefficient** | **Hazard Ratio** | **Standard Error** | **Z Value** | **P Value** | **95% CI Lower** | **95% CI Upper** |
| --- | --- | --- | --- | --- | --- | --- | --- | --- | --- |
| Active solid neoplasia | 149 | 45 | 0.079 | 1.082 | 0.164 | 0.485 | 0.627 | 0.785 | 1.493 |
| Active hematological neoplasia | 31 | 16 | 0.682 | 1.979 | 0.259 | 2.626 | 0.008 | 1.189 | 3.295 |
| Active autoimmune disease | 57 | 8 | -0.665 | 0.514 | 0.359 | -1.849 | 0.064 | 0.254 | 1.040 |
| Active autoimmune disease with treatment | 37 | 4 | -0.966 | 0.380 | 0.503 | -1.917 | 0.055 | 0.141 | 1.021 |
| Use of immunosuppressants | 100 | 34 | 0.237 | 1.268 | 0.184 | 1.287 | 0.197 | 0.883 | 1.820 |
| Use of immunosuppressants in patients with HIV | 10 | 3 | 0.092 | 1.096 | 0.586 | 0.157 | 0.874 | 0.347 | 3.464 |
| Use of immunosuppressants due to chemotherapy in the last 30 days | 23 | 12 | 0.658 | 1.932 | 0.300 | 2.189 | 0.028 | 1.071 | 3.484 |
| Chronic use of corticosteroids | 43 | 12 | -0.054 | 0.946 | 0.295 | -0.184 | 0.853 | 0.530 | 1.690 |
| Chronic use of other immunosuppressants | 28 | 8 | 0.076 | 1.079 | 0.359 | 0.213 | 0.831 | 0.533 | 2.183 |
| Bone marrow transplant | 6 | 4 | 1.368 | 3.928 | 0.508 | 2.691 | 0.007 | 1.450 | 10.639 |
| Neutropenia associated with immunosuppression | 4 | 2 | 0.468 | 1.598 | 0.718 | 0.652 | 0.514 | 0.390 | 6.540 |
| AIDS | 4 | 2 | 0.882 | 2.417 | 0.716 | 1.231 | 0.218 | 0.592 | 9.853 |
| Induction to immunosuppression | 19 | 6 | 0.043 | 1.044 | 0.414 | 0.105 | 0.916 | 0.463 | 2.355 |
| Chemotherapy | 49 | 19 | 0.405 | 1.500 | 0.240 | 1.688 | 0.091 | 0.936 | 2.401 |

**Supplementary Table 5.** Adjusted Association Between Nosocomial Lower Respiratory Tract Infections (nLRTI), Immunosuppression Status, and In-Hospital Mortality, Stratified by nLRTI Subtype.

| **Variable** | **VAP (n=555)** | | **VAT (n=160)** | | **HAP (n=152)** | | **ICUAP (n=98)** | | **VHAP (n=94)** | |
| --- | --- | --- | --- | --- | --- | --- | --- | --- | --- | --- |
|  | **OR (IC 95%)** | **p-value** | **OR (IC 95%)** | **p-value** | **OR (IC 95%)** | **p-value** | **OR (IC 95%)** | **p-value** | **OR (IC 95%)** | **p-value** |
| **Immunosuppression** | 1.70 (1.10–2.62) | 0.016 | 1.10 (0.48–2.55) | 0.819 | 1.22 (0.60–2.48) | 0.582 | 0.93 (0.34–2.56) | 0.889 | 1.88 (0.67–5.26) | 0.227 |
| **Age** | 1.03 (1.02–1.04) | <0.001 | 1.01 (0.99–1.03) | 0.218 | 1.00 (0.98–1.03) | 0.712 | 1.02 (0.98–1.05) | 0.296 | 0.99(0.96–1.02) | 0.805 |
| **SAPS II** | 1.02 (1.00–1.03) | 0.006 | 1.01 (0.99–1.03) | 0.334 | 1.04 (1.02–1.06) | 0.001 | 1.02 (0.99–1.05) | 0.162 | 1.05 (1.02–1.09) | 0.004 |
| **Pseudo R²** | 0.0596 | — | 0.0193 | — | 0.0806 | — | 0.0327 | — | 0.0838 |  |
| **Abbreviations**: nLRTI, Nosocomial lower respiratory tract infection, VAP: Ventilator-associated pneumonia, HAP: Hospital-acquired pneumonia, VAT: Ventilator-associated tracheobronchitis, ICUHAP: Hospital-acquired pneumonia in the ICU, VHAP: Ventilated hospital-acquired pneumonia | | | | | | | | | | |
